# P-1939. Health status and symptom perception among patients recently diagnosed with Long COVID in the United States

**DOI:** 10.1093/ofid/ofae631.2098

**Published:** 2025-01-29

**Authors:** Thomas F Oppelt, Michael Caron, Jamie Zagorski, Rodney H Taylor, Chinonso Akano, Lisa McCorkell, LaKeisha Williams, Gary A Puckrein

**Affiliations:** Gilead Sciences, Inc, Foster City, California; Gilead Sciences, Inc., Foster City, California; Gilead Sciences, Inc., Foster City, California; Gilead Sciences, Inc., Foster City, California; Gilead Sciences, Inc., Foster City, California; Patient-Led Research Collaborative, Oakland, California; Center for Minority Health and Health Disparities Research and Education, Xavier University of Louisiana, New Orleans, Louisiana; National Minority Quality Forum, Washington, District of Columbia

## Abstract

**Background:**

Long COVID (LC) remains an urgent public health concern. The objective of this study was to explore recent patient perspectives of the COVID-19– or LC–related journey. Perceptions regarding symptoms and health status among patients diagnosed with LC are reported.

**Figure:**
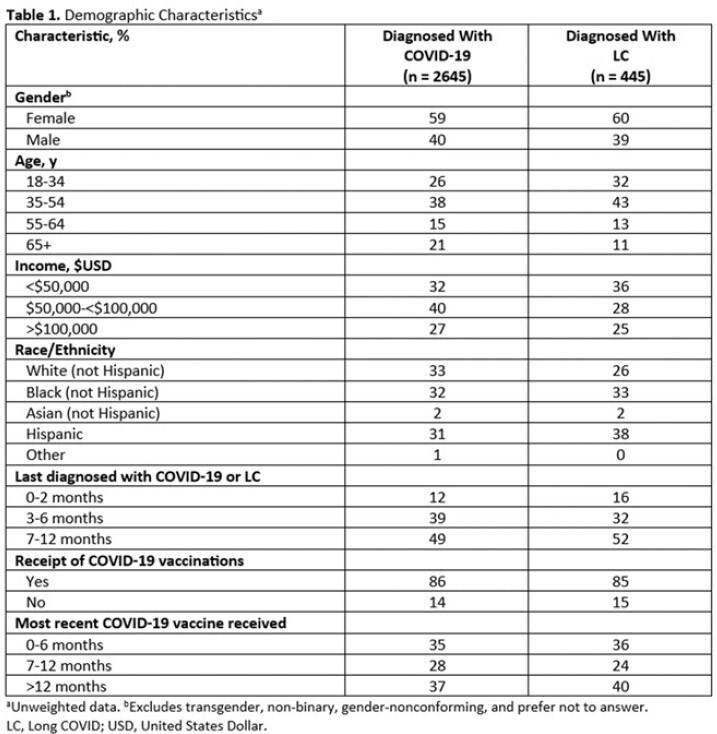

**Methods:**

An online survey study was conducted in the United States by the Harris Poll (March 12-April 1, 2024) in 3090 adults aged ≥18 y who had COVID-19 or LC diagnosed in the previous 12 months. Non-demographic data were weighted by age, gender, race/ethnicity, region, education, marital status, household size, household income, and propensity to be online. Sampling precision was measured using a Bayesian credible interval. Statistical significance was determined by two-tailed t-test at the 95% (*P*< 0.05) confidence level.

**Figure:**
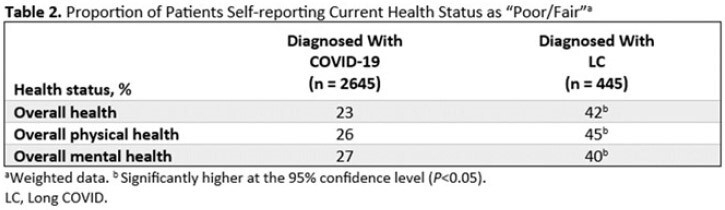

**Results:**

Of respondents, 2695 were diagnosed with COVID-19 and 445 were diagnosed with LC in the previous 12 months. Demographics were generally similar between groups (Table 1). A significantly greater proportion of LC patients (42%) self-described their current overall health as poor/fair vs those with COVID-19 alone (23%, *P*< 0.05; Table 2); LC patients also reported poorer physical and mental health. LC patients were significantly more likely to describe their initial LC symptoms as very/somewhat severe compared to the initial COVID-19 symptoms described by patients who did not develop LC (69% vs 44%, respectively; *P*< 0.05). Figure 1 shows self-reported COVID-19 or LC symptoms experienced within the first 3 days of feeling sick. LC patients waited over twice as long as those who did not develop LC to seek medical care following initial symptoms (8.0 vs 3.2 days, respectively; *P*< 0.05); Hispanic patients reported the longest wait times. Overall, 57% of respondents diagnosed with LC had experienced symptoms for ≥1 year. Fatigue and headache were reported as the most severe persistent symptoms (Figure 2).

**Figure:**
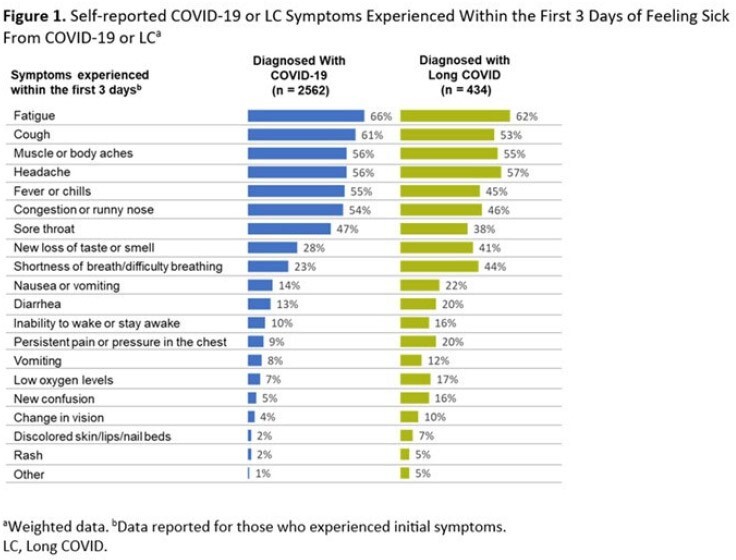

**Conclusion:**

COVID-19 and LC remain important areas of concern for the US population. Patients with recent LC reported greater initial LC symptom severity, a longer time to seeking medical care, and poorer overall, physical, and mental health compared with those who did not develop LC. Further analyses will explore perspectives related to healthcare access and differences by race/ethnicity.

**Figure:**
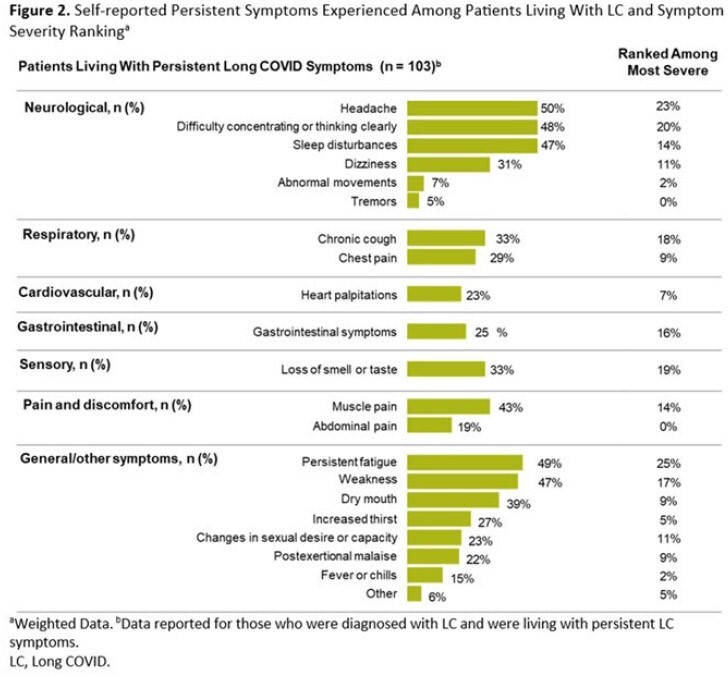

**Disclosures:**

Thomas F. Oppelt, PharmD, BCPS, Gilead Sciences, Inc: I am an employee of Gilead Sciences, Inc|Gilead Sciences, Inc: Stocks/Bonds (Public Company) Michael Caron, PharmD, Gilead Sciences, Inc.: Employee|Gilead Sciences, Inc.: Stocks/Bonds (Public Company) Jamie Zagorski, MSN, FNP, Gilead Sciences, Inc.: Employee|Gilead Sciences, Inc.: Stocks/Bonds (Public Company) Rodney H. Taylor, PharmD, Gilead Sciences, Inc.: Employee|Gilead Sciences, Inc.: Stocks/Bonds (Public Company) Chinonso Akano, PharmD, Gilead Sciences, Inc.: Employee|Gilead Sciences, Inc.: Stocks/Bonds (Public Company) LaKeisha Williams, PharmD, MSPH, Gilead Sciences, Inc.: Grant/Research Support Gary A. Puckrein, PhD, Dexcom: Grant/Research Support|Gilead Sciences, Inc.: Grant support for National Minority Quality Forum|National Minority Quality Forum: Employee

